# Sequence Capture of Mitochondrial Genome with PCR-Generated Baits Provides New Insights into the Biogeography of the Genus *Abies* Mill.

**DOI:** 10.3390/plants11060762

**Published:** 2022-03-13

**Authors:** Vladimir L. Semerikov, Svetlana A. Semerikova, Yuliya Y. Khrunyk, Yuliya A. Putintseva

**Affiliations:** 1 Institute of Plant and Animal Ecology, Ural Branch of the Russian Academy of Sciences, 620144 Ekaterinburg, Russia; s.a.semerikova@ipae.uran.ru; 2 Department of Heat Treatment and Physics of Metal, Ural Federal University Named after the First President of Russia B.N. Yeltsin, 620002 Ekaterinburg, Russia; juliakhrunyk@yahoo.co.uk; 3 Department of Biophysics, Institute of Fundamental Biology and Biotechnology, Siberian Federal University, 660041 Krasnoyarsk, Russia; yputintseva@sfu-kras.ru

**Keywords:** intercontinental migrations, mitochondrial DNA, target enrichment, phylogeny, molecular dating

## Abstract

Mitochondrial DNA (mtDNA), being maternally inherited in plants of the family Pinaceae, is an important source of phylogeographic information. However, its use is hindered by a low mutation rate and frequent structure rearrangements. In the present study, we tested the method of genomic libraries enrichment with mtDNA via the sequence capture method yielding mtDNA data which were further used to reconstruct the phylogenetic tree of the genus *Abies*. The baits for hybrid capture were obtained by long-range PCR using primers designed on the basis of the assembly of *Abies sibirica* Ledeb. mitochondrial genome. Mitochondrial genomes of *Picea sitchensis* (Bong.) Carr., *Larix sibirica* Ledeb., and *Keteleeria davidiana* (Bertrand) Beissn. were used as an outgroup. The resulting phylogenetic tree consists of two sister branches, including the Eurasian and American species, respectively, with some exceptions. The subclade of *A. sachalinensis* (F. Schmidt) Mast. and *A. veitchii* Lindl. (Japan and Sakhalin islands) occupies a basal position in the branch of American firs, probably due to the complex history of fir migrations from North America to Eurasia. The tree has high support for majority of clades. For species represented by more than one sample an intraspecific variability was found which is suitable to design mtDNA markers for phylogeographic and population studies.

## 1. Introduction

DNA of genomes inherited maternally and transmitted through seeds has a reduced mobility in a succession of generations. Hence, the spatial structure of its variability is more slowly erased by the gene flow and better preserves information about the history of the species (migration, refugia, expansion and contraction of the range) than the DNA transmitted through pollen [1]. In addition, in the history of various taxonomic groups, the displacement of some related species by others is often accompanied by introgressive hybridization and the capture of maternally inherited genomes of the displaced species by the invading ones [2], which is revealed in the reticulate evolution and in conflict between phylogenetic trees of DNA of maternally and paternally inherited organelles or nuclear DNA [3,4]. Thus, their comparison can provide information about both the initial colonization by seeds and subsequent migrations associated with pollen transfer and introgressive hybridization. These circumstances determine the importance of using several genomes, including those with maternal inheritance, for biogeographic research. In species of the family Pinaceae, mtDNA has maternal inheritance [5]. However, the availability of mitochondrial DNA markers is limited by a number of factors, i.e., the lack of variable “universal” regions [6], a low variability of mtDNA due to a low mutation rate [7] and a high rate of rearrangements [8] of the plant mitochondrial genome, which hinders the identification of homologous regions of the genome even in related species. Though progress in next-generation sequencing techniques can overcome or compensate for these challenges, genome-wide sequencing of multiple samples of coniferous species having a genome size of about 10–30 × 10^9^ bp is still not feasible. An alternative to the whole genome sequencing is the use of genomic libraries enriched with mtDNA via the target-enrichment approach [9].

**Figure 1:**
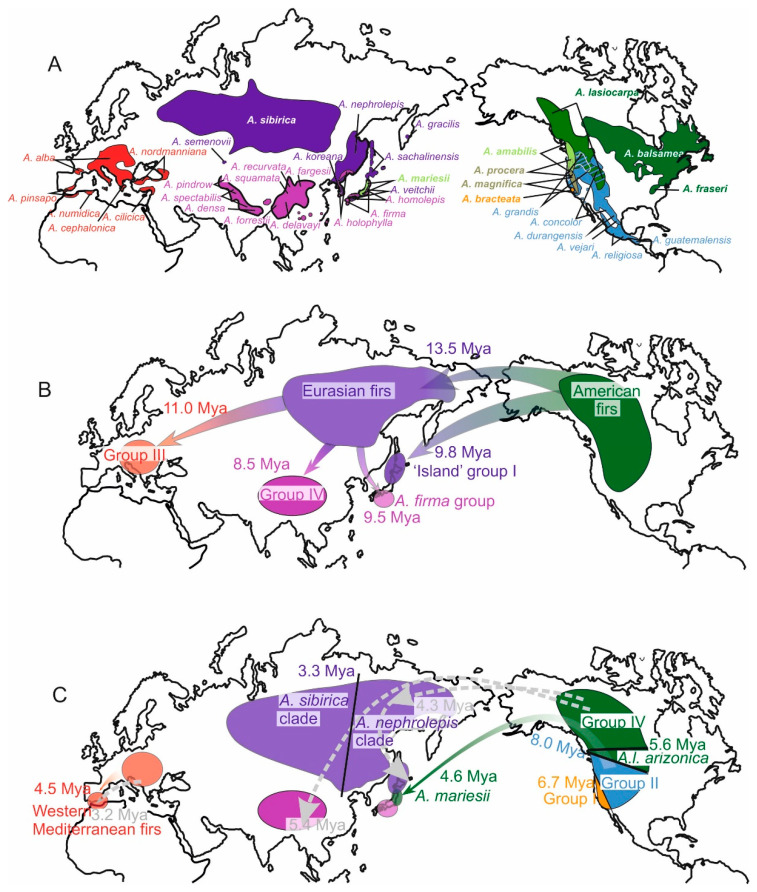
Distribution and colonization history of *Abies* species, suggested on the basis of mtDNA phylogeny (this study) and taking into account the results of biogeographic study based on nuclear DNA data [10]. The events of migration and isolation are presented with an age estimate. Recent and ancestral taxa are color-coded according to the nuclear DNA-based groups [10]. Additionally, the proposed migrations associated with introgressive hybridization and capture of mitochondrial genome of native species by migrants are indicated (dashed gray line). (**A**) Present species ranges; (**B**) Before 8 million years ago (Mya); (**C**) After 8 Mya years ago.

*Abies* (firs) is one of 11 genera of the family Pinaceae, the most important forest-forming taxonomic group in the Northern Hemisphere (Figure 1).

Previous biogeographic studies based on nuclear, chloroplast and mitochondrial DNA pointed to the American origin of modern *Abies* lineages and multiple migrations from North America to Eurasia [10,11] (Figure 1B,C). Additionally, it was shown that, in contrast to nuclear and chloroplast DNA, mitotypes of firs form two groups consisting of Eurasian and (predominantly) American species, probably due to the origin of mitochondrial genomes of Eurasian firs being the result of primary colonization from North America. Presumably, during subsequent migrations from North America, migrant species captured the mitochondrial genomes of aboriginal species through introgressive hybridization which explains the presence of phylogenetically related mitotypes in unrelated groups of Eurasian species. The mitotype tree, however, had a low resolution. In particular, the main groups of mitotypes within the American and Eurasian branches were not supported, and the position of the outgroup was not resolved.

The aims of this study are: (1) based on a well-sampled *Abies* taxa and enlarged mtDNA dataset to resolve evolutionary relationships among species and infer timings of diversification based on mtDNA and (2) to propose an approach for identifying mtDNA polymorphism within and among closely related conifer species in order to develop markers that are suitable for phylogeographic, population, and applied research. For this purpose, we used NGS sequencing of genomic libraries of the majority of *Abies* species enriched with mtDNA. Baits for hybrid capture with a total length of about 800,000 bp were prepared using long-range PCR and primers designed based on the assembly of Siberian fir mitochondrial genome.

## 2. Results

Assembly of A. sibirica genome consisted of 4,547,516 contigs longer than 200 bp with a total length of 2.39 Gbp, N50  =  639 bp and the longest contig 163,323 bp. Due to low coverage, the resulting whole genome assembly was fragmented, but for the purposes of our study, it was enough to identify mitochondrial contigs. The BLAST [12] search against all mitochondrial plant sequences indicated 19 contigs as mitochondrial with total length of 906,223 bp. The largest contig was 163,323 bp long. A set of 39 protein coding genes has been identified: *atp4*, *atp6*, *atp8*, *atp9*, *ccmB*, *ccmC*, *ccmFc*, *ccmFn*, *cob*, *cox1*, *cox2*, *cox3*, *matR*, *mttB*, *nad1*, *nad2*, *nad3*, *nad4*, *nad4L*, *nad5*, *nad6*, *nad7*, *nad9*, *rpl2*, *rpl5*, *rpl16*, *rps1*, *rps2*, *rps3*, *rps4*, *rps7*, *rps10*, *rps11*, *rps12*, *rps13*, *rps14*, *rps19*, *sdh3* and *sdh4*. Only 11 tRNA genes were discovered. This may be due to the fact that tRNAs are short (less than 100 bp) and, as a result, contigs containing them are poorly detected by the BLAST if they do not contain other mitochondrial genes. A single rRNA gene *rrn18* was also found.

In total, 96 genomic libraries were enriched with mtDNA and sequenced. The resulting reads were mapped to the reference, i.e., concatenated contigs of Siberian fir mitochondrial genome. Overall, 9 of the 96 samples mapped to the reference had an average coverage below 20, probably due to technical errors during the preparation of the libraries, and, thus, were excluded from further analysis. The rest of the samples were sequenced successfully, although the coverage among them varied significantly: the minimum ratio of the number of scored nucleotides to the length of the alignment was 27% and the maximum ratio accounted for 92%. Coverage significantly depended on the phylogenetic distance from the reference being the highest in A. sibirica and related firs of North Asia (84%), and noticeably lower in other East Asian species (60%), while in Mediterranean, American, as well as Japanese firs related to the American ones it accounted for 53%. The length of the alignment, including the outgroup, was 918,895 bp. Among the Abies, 12,293 SNPs and 3533 indels were identified. The coding sequences, including exons, ribosomal RNAs and transfer RNAs, were 37,370 bp (Table S2) in alignment and contained only 113 SNPs and 3 indels.

### 2.1. MtDNA Tree

**Figure 2:**
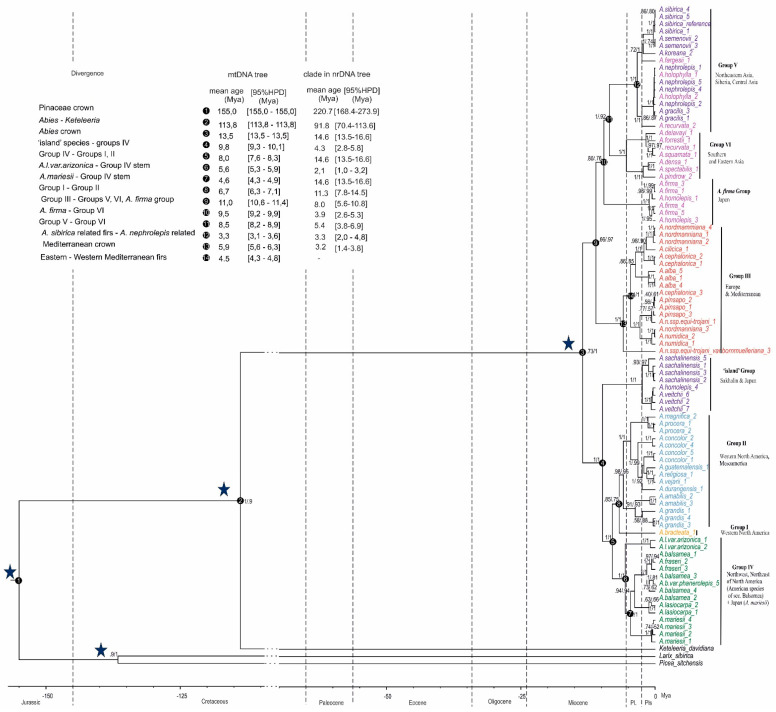
Dated phylogeny obtained from treePL using penalized likelihood and fossil constraints and based on the maximum likelihood tree generated from RAxML. Values above branches represent ML posterior probabilities/MP bootstrap support. The stars indicate the nodes, calibrated by the fossils (see Material and Methods). The dashed lines mark the boundaries between geological epochs (Pliocene and Pleistocene are abbreviated as Pl. and Pls., respectively). The taxon names are colored according to Figure 1. Numbers following taxon names refer to taxon sample numbers (Table S1).

ML and MP phylogenetic trees were nearly identical (Figure 2 and Figure S1) and consisted of two sister clades formed almost exclusively by Eurasian and American species.

The American clade was formed by three subclades: the first one included the mitotypes of insular Asiatic firs, i.e., *A. sachalinensis, A. veitchii* and a single mitotype of *A. homolepis* Siebold & Zucc. The latter is not unusual given the presence of shared mitotypes in Japanese fir species [13]. This subclade was sister to the pair of others: the second subclade consisting of American firs of section *Balsamea* plus the Japanese fir *A. mariesii* Mast. which is a sister taxon to the American fir *A. amabilis* Douglas ex J. Forbes and the third subclade comprising the rest of American species.

Mediterranean species formed the most basal group of the Eurasian clade with subsequent sequential separations of the group of mitotypes belonging to the Japanese species *A. firma* Siebold & Zucc. and *A. homolepis*, the group of South Asian species mitotypes and the group of the mitotypes of the Northeast Asia species including Siberian fir. Most of the clades were highly supported, but for some of them the support was moderate (Figure 2). The low posterior probability of the *Abies* clade (73%) is probably linked to the low coverage in *Keteleeria davidiana*, while the low posterior probability of the Eurasian clade (66%) and Asian subclade (80%) could have resulted from the incomplete lineage sorting and recombination at the beginning of the *Abies* divergence, which led to a random distribution of ancestral characters in the basal lineages. The ML tree constructed without outgroup taxa showed a much higher support for the Eurasian clade and the Asian subclade (85% and 98%, respectively), which is consistent with these assumptions.

### 2.2. Possible Admixtute among the Fir Lineages

The isolated position of the group of mitotypes belonging to the island species *A. sachalinensis* and *A. veitchii* was unexpected. According to nuclear DNA data [10], these species fall into the group of Asian species of section *Balsamea* (including *A. sibirica, A. nephrolepis* (Trautv. ex Maxim.) Maxim. and *A. koreana* E. H. Wilson) which is sister to the group of American species of this section (*A. balsamea* (L.) Mill., *A. lasiocarpa* (Hook.) Nutt., *A. lasiocarpa var. arizonica* (Merriam) Lemmon, etc.) with the age of split between American and Eurasian Balsamea being estimated at 4.3 Mya. Presumably, during multiple migrations of firs from North America to Asia and as a result of introgressive hybridization, migrant species captured the mtDNA of the species which had already inhabited Asia, although some of them (*A. sachalinensis*, *A. veitchii* and *A. mariesii*) retained their ancestral (American) mtDNA [10], probably due to the isolation on the Sakhalin and Japan islands. While adopting this hypothesis, one should expect that the mtDNA of the island group (*A. sachalinensis* and *A. veitchii*) should be related to the mtDNA of the American species of section *Balsamea*, but this was not the case judging from the mitochondrial tree (Figure 2) and the island group appeared to be the most basal in the American branch. On the other hand, previous studies of the mtDNA of the Sakhalin fir revealed the chimeric mitotypes, combining the traits of both the American species and neighboring Asian species, which might have been the result of mtDNA recombination [10].

Consequently, the isolated position of insular species within the American clade can be explained by an admixture of mtDNA from Asian species. We used several analyses to test this assumption. First, we compared five main groups of Eurasian species identified in the phylogenetic tree: Mediterranean (1), South Asian (2), species related to *A. sibirica* (3), Northeast Asian (4) and *A. firma* group (5) as well as three groups of species belonging to American clade: island group (6), American firs of section *Balsamea* together with the Japanese fir *A. mariesii* (7) and the rest of American species (8) with each other (Table S2). It was shown that among three American groups, the island one turns out to be the closest to all five Eurasian groups in terms of both the number of nucleotide differences and the number of indel differences. This observation supports the assumption that a significant proportion of the mitochondrial genome of island group is of Asian origin. We further verified the hypothesis regarding the introgression using the ABBA-BABA approach. We found 32 mutations (indels and SNPs) common to the consensus of the Eurasian clade (3) and the consensus of island species (2) and satisfying the condition that the consensus of the remaining American species (1) and the consensus of the outgroup (4) had the ancestral state of these traits (combination ABBA). However, 62 mutations common to 1 and 3 were also found while 2 and 4 had the ancestral state (BABA), i.e., this test did not detect the admixture. Thus, there was no clear indication of whether the introgression of mitochondrial genes of Asian species is the cause of the divergent position of island species in the group of American firs.

## 3. Discussion

### 3.1. Sequence Capture Data Shed New Light on the Evolution and History of the Colonization of Abies through Seed Dispersion

A phylogenetic study of the genus *Abies* based on a significant part of the mitochondrial genome confirmed the deep divergence of *Abies* species into the American and Eurasian clades and the origin of the latter as a result of a single migration from America (Figure 1 and Figure 2). In a study carried out by [10], based on three fragments of mtDNA, a network of *Abies* mitotypes were divided into Eurasian and American branches. However, they had little support and the position of the root was not determined. In a recent research study [14], resequencing of the mitochondrial contigs of Siberian fir in fifteen species, representatives of the main phylogenetic groups of firs, and *Keteleeria*, and with a total length of alignment of about 30,000 nucleotides, showed that the mitotypes of fir were also divided into European and American groups, which did not have a sister relationship as the Eurasian group fell into the American one, albeit with weak support.

In the present study, although based on substantially more data, the position of the root and the sister relationship of the Eurasian and American groups were also not highly supported. In addition to low coverage in the *Keteleeria*, this may be due to incomplete lineage sorting in combination with the recombination of mtDNA lineages, which could have led, at the beginning of the crown group divergence, to the formation of random combinations of ancestral and derived traits in different lineages.

The assessment of the age of the crown group of firs (13.5 Mya, Figure 2) by means of mtDNA, is consistent with the estimate based on nuclear DNA (15 Mya) [10]. However, for branches whose positions in mitochondrial and nuclear DNA trees are different, the estimates of the divergence time differ as well. For example, on mtDNA tree the spit age of the island group from other American species (9.7 Mya, Figure 2) is much older than the respective age on nuclear DNA tree being 4.3 Mya [10]. The *A. firma*-*A. homolepis* group is a sister to the group of South Asian species and the age of split between them is estimated as 3.9 Mya according to nuclear DNA, though based on mtDNA the former group is sister to all remaining Asian species with a split age of 9.5 Mya. Some clades, although having been recognized in both the nuclear and mitochondrial trees, point to significantly different ages of differentiation. For example, on the mtDNA tree, the age of differentiation of the Mediterranean group is 5.9 Mya in the mitochondrial tree and 3.2 Mya based on nuclear markers. Interestingly, this age was even lower for chloroplast DNA, i.e., 1.2 Mya [10]. Most probably, these differences are due to the mode of inheritance of the three genomes and the associated level of the gene flow: the gene flow of chloroplast and nuclear DNA, caused by the transfer of pollen, promotes fixation of polymorphism more efficiently than the gene flow through the maternally inherited and solely seed-transmitted mtDNA [15]. The geographic barriers between Mediterranean firs could have been overcome by pollen flow more often than through seed migration. It is interesting that in a study of [16], based on RAD-seq data, the age of differentiation of Mediterranean firs corresponded to the Oligocene–Miocene boundary, which is much higher than our estimates. We believe that this is due to a controversial approach to calibration of the tree in [16] where the age of the oldest *Abies* fossil [17] was treated as a minimum age of calibration of the root by applying a log-normal distribution of 47 Mya to the age of the oldest node.

Both the isolated position of the island firs in the mtDNA tree (Figure 2) and the age of separation from other American clades significantly contradict the previous assumption that the island species retain mtDNA inherited from their American ancestor [18]. At the same time, our hypothesis that significant difference in island species mtDNA from the mtDNA of other American firs resulted from the introgression of genes from Asian firs is not supported by the ABBA-BABA test.

According to the alternative hypothesis, the mtDNA of the island species was not obtained from the American ancestors, but, similar to other Asian species, the island species captured mtDNA of firs that had invaded Eurasia earlier. Probably, during the migration of the firs of Balsamea section from North America, the species with American-type mtDNA had already inhabited Sakhalin and the Japan islands. The ancestor of modern *A. sachalinensis* and *A. veitchii* displaced them, acquiring their mtDNA as a result of introgressive hybridization (as illustrated in Figure 1B,C).

Samples of most species form monophyletic clades (Figure 2), though variation was observed within the species that were represented in the analysis by more than one accession. Among the 4 *A. sibirica* sequences, including the reference, 16 mutations were encountered, 4 of which were previously detected during resequencing of *A. sibirica* mitochondrial contigs and were used in phylogeographic research [19]. In 3 *A. alba* Mill. accessions, 33 intraspecific polymorphisms were found. Most of them indicate the differences between the samples from Austria and from Poland. *A. concolor* (Gordon) Lindl. Ex Hildebr. showed two deeply diverged clades differing from each other by 150 mutations: the first clade includes two accessions from Yosemite and, according to geographical origin, belongs to the variety *Abies concolor var. lowiana* (Gordon) Lemmon, and the second consists of specimens of unknown origin and is close to other Mesoamerican firs. In total, 6 samples of *A. balsamea* (including *A. balsamea var. phanerolepis* Fernal and *A. fraseri* (Pursh) Poir.) contained 119 polymorphisms. The revealed polymorphisms can be used to develop mtDNA markers for many *Abies* species. Earlier Donnelly et al., (2017) [20] showed the possibility of using whole genome sequencing data to design mtDNA markers for pines, i.e., *Pinus sylvestris* L. and several related species. For this purpose, whole genomes of several samples were sequenced with low coverage. In our method, due to the use of the “sequence capture” approach, mainly mtDNA and a smaller proportion of nuclear and chloroplast DNA were sequenced which increases the efficiency of the ILLUMINA use and allowed partial sequencing of the mitochondrial genomes consisting of 87 representatives of most of *Abies* species.

### 3.2. Solving the Problem of Sample Classification Errors

Closely related species in conifers usually have few diagnostic morphological characters, contributing to confusion in botanical garden collections, which is a major problem for phylogenetic studies. Correcting classification errors in the material used requires the inclusion of controversial specimens in a phylogenetic study using different genetic markers. However, for reliable recognition of related species, chloroplast genetic markers are often not sufficiently variable between species, nuclear markers are too variable within species, and mitochondrial markers are too few. The method used makes it possible to sequence a significant part of the mitochondrial genome, which increases the possibility of identifying and correcting classification errors. A well-supported topology has been found in Mediterranean firs (Figure 2). Among them, a specimen *A. nordmanniana* ssp. *equi-trojani var. burnmulleriana* (Mattf.) Silba was the most divergent. The remaining accessions were divided into two clades, the first of which included a subclade of *A. numidica* de Lannoy ex Carriere accessions plus one specimen of *A. nordmanniana* (Steven) Spach. from the Sochi Botanical Garden (*A. nordmanniana*_3) and a subclade that included *A. pinsapo* Boiss. accessions plus *A. cephalonica* Loudon individual (*A. cephalonica*_3) from the Kornik Botanical Garden and a sample of *A. nordmanniana* ssp. *equi-trojani* (Asch. & Sint.ex Boiss.) Coode &Cullen (*A. n.* ssp. *equi-trojani*_1) from the Nikitsky Botanical Garden. The second clade consisted of the *A. alba* subclade, the *A. cephalonica* subclade and the *A. nordmanniana* plus *A. cilicica* (Antoine & Kotschy) Carrière subclade. Unusual clustering of the three above samples was also previously observed in a study based on AFLP data [21], in which the *A. nordmanniana*_3 was grouped with *A. numidica* specimens, and the other two ones were clustered with *A. pinsapo*, which strengthens the assumption that they had been misclassified or of hybrid origin. If errors were made in the classification of three specimens: *A. nordmanniana*_3, *A. cephalonica*_3, and *A. n.* ssp. *equi-trojani* _1, and they belonged to *A. numidica* and *A. pinsapo*, then, firstly, the investigated Mediterranean samples would have been clustered strictly according to taxonomy, and secondly, the western group of species (*A. pinsapo* and *A. numidica*) would have been monophyletic with mtDNA.

### 3.3. Pros and Cons of the Used Approach

The size of the resulting assembly of the mitochondrial genome of *A. sibirica* (906,223) is somewhat inferior to the size of the published genomes of other plants of the Pinaceae family: *Picea sitchensis*: 5,520,000 bp [22], *Larix sibirica*: 11,300,000 [23], *Pinus taeda*: 1,191,000 [24] (Kan 2020) and *P. abies*: 4,899,000 [25]. It is also inferior to the above species in the number of protein-coding genes: 39 versus 41, 40, 41 and 41, respectively, and in the number of identified tRNAs: 11 versus 27, 34, 12 and 17, respectively. This is quite expected given that obtaining the entire mitochondrial genome of *A. sibirica* was not the aim of the sequencing project [26]. However, this assembly turned out to be quite sufficient for the development of baits and as a reference for genome mapping, and, ultimately, to obtain a well-resolved phylogenetic reconstruction. MtDNA is an important source of biogeographic information for coniferous plants. Moreover, conserved sequences of plant mitochondrial genome are suitable for deep phylogenetic reconstructions [27,28,29]. However, the low rate of nucleotide substitutions (e.g., [7,30]) and the dynamic nature of plant mtDNA structure [31] complicate its use. Next-generation sequencing in combination with “target sequence capture” is an efficient approach to obtain a sufficient amount of phylogenetic data for both nuclear and organelle genomes ([32] for example). This method was used to study the dynamics of mixed populations of *Larix sibirica*-*Larix gmelinii* (Rupr.) Rupr. at the polar tree line in Eastern Siberia using ancient chloroplast DNA from Holocene lacustrine sediments [33]. However, to our knowledge, it has not been used for studies of conifers based on mitochondrial genomes. We have adapted the existing techniques to obtain a large amount of data on the variability of mtDNA in many species within the genus. The method uses mostly inexpensive reagents, consumables and common equipment found in most laboratories. Instead of expensive RNA-based baits, we used PCR-amplified ones [34]. BLAST selection of contigs of mitochondrial origin among contigs of the Siberian fir genome assembly provided the experiment with baits and with a reference, mapping of short reads to which excludes data contamination by chloroplast and nuclear sequences. An additional evidence of the absence of a significant admixture of non-mtDNA is the extremely low nucleotide diversity: 1.5 variable nucleotides per 100 nucleotides of alignment, compared to 9.2 in 10 nuclear fragments in a previous phylogenetic study [10].

At the same time, the proposed approach has significant limitations. First, for the development of baits and for mapping of NGS reads, a sufficiently high-quality reference mitochondrial genome for the genus is required. Fortunately, this problem should gradually disappear thanks to the ever-increasing number of complete genome sequences in databases. Second, due to the high rate of mtDNA rearrangements, it is difficult to obtain data on more than one genus, and a common alignment from representatives of different genera can be constructed probably only for a limited fraction of the mitochondrial genome. This limitation can be partially overcome by employing mitochondrial genomes of several distantly related taxa for developing baits, which was shown in the study of [35], where a bait set for nuclear loci of 17 Lepidoptera families was designed.

## 4. Materials and Methods

**Figure 3:**
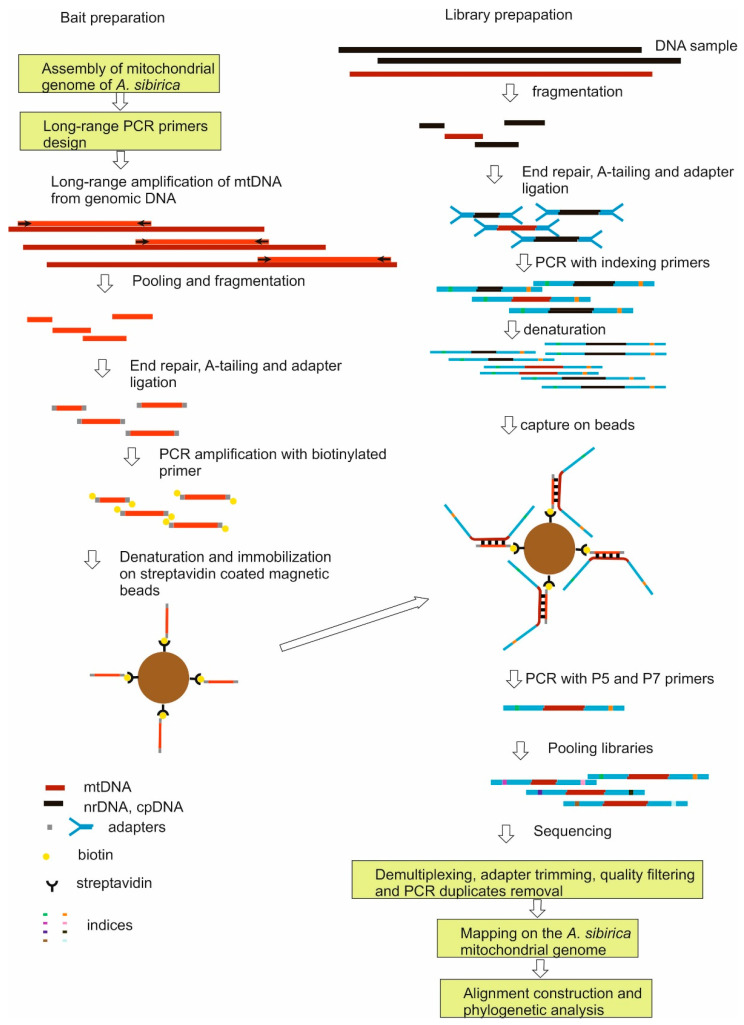
Overview of sequence capture method. The left side shows the preparation of baits, the right side shows the preparation of the indexed library, NGS sequencing of the combined individual libraries, bioinformatics analysis of the sequencing results. Genomic DNA is shown in black and dark red (mtDNA), bytes in light red, adapters and PCR primers in gray and cyan, indices in other colors.

Genomic libraries were prepared from 95 DNA samples of 42 *Abies* taxa out of 47 species of fir identified by [36], representing all major phylogenetic lineages and previously used in a phylogenetic study [10] and a sample of *Keteleeria ortune* (A. Murray) Carrière (Table S1). To enrich the libraries by mtDNA, the target-enrichment strategy with hybrid capture was used [9] as shown in the diagram (Figure 3). Laboratory procedures were based on techniques of [34,35,37,38]. Their detailed description is presented in the Laboratory Protocols (Supplementary Materials).

### 4.1. Assembly of Abies Sibirica Genome and Mitochondrial Contigs Selection

We assembled the Abies sibirica genome using the data from whole genome sequencing of the Abies sibirica tree of unknown origin, which was used early [26] (ENA accession no. ERP002568). The whole genome sequencing data contained 123.3 Gbp in 616.5 million reads (SRA accession ERX242659), yielding 8-fold coverage of the roughly 15.5 Gbp nuclear genome [39]. The quality of Illumina reads was assessed using FastQC v. 0.11.5 [40]. Adapter sequences were trimmed and short reads were filtered using Trimmomatic v. 0.36 [41] with minimum quality of 20 and minimum length of 35 bp. The obtained paired-end sequence reads were assembled de novo into contigs using the CLC Assembly Cell v. 5.0.0. The BLAST search against all mitochondrial plant sequences available in the NCBI GenBank and other publicly available databases was used to mine mitochondrial contigs from this assembly. Draft annotation of the mitochondrial genes was carried out on the basis of homology with protein-coding sequences of Ginkgo biloba, Cycas taitungensis and Larix sibirica. tRNA genes were discovered using ARAGORN [42] and tRNAscan-SE 2.0 online tool with default parameters [43]. Ribosomal RNA (rRNA) was annotated using RNAmmer [44].

### 4.2. Preparing of Baits

The baits were prepared from the PCR-amplified fragments of mtDNA of *Abies sibirica*. PCR primers were designed using the 18 longest of the above contigs. The long-range PCR via LongAmp^®^ Taq DNA Polymerase (New England Biolabs Inc., Ipswich, Massachusetts, USA ) was employed, allowing the use of a minimum number of primer pairs (81) and PCR reactions. Primers (Laboratory Protocols, Supplementary Materials) were designed using the Primer 3 (v. 0.4.0) program [45] assuming that the PCR fragment size is 10,000–12,000 bp with a minimal overlapping of PCR products. Their total length was about 800,000 bp. The quality and the relative amount of the PCR product in each reaction were checked visually on agarose gel. Most of the PCR fragments were amplified successfully and the PCR products did not contain a substantial amount of non-specific amplification. PCR products were combined in equimolar concentrations and cleaned with SPRI beads using the CleanMag kit (Evrogen, Moscow, Russia). The mixture of PCR products was treated with NEBNext dsDNA Fragmentase (New England Biolabs) to obtain DNA fragments with an average size of about 400 bp. Shorter and longer DNA fragments were removed using the dual fragment size selection method (DFSS) [38]. Following the end-repair and A-tailing, fragments were ligated to adapters and amplified with biotinylated primer (Laboratory Protocols, Supplementary Materials).

### 4.3. Preparation of Libraries

About 300 ng (or less) of DNA from each sample (Table S1) was fragmented with NEBNext dsDNA Fragmentase and fractionated using the DFSS method to obtain DNA fragments with an average size of about 400 bp. Following the end-repair and A-tailing, the fragments were ligated to adapters prepared by annealing of the NEBNextad1 and NEBNextad2 oligonucleotides (Laboratory Protocols, Supplementary Materials) and amplified individually with the NEBNext i50x and NEBNext i7yy primer pairs containing 8-character indices and using the minimum number of cycles (Laboratory Protocols, Supplementary Materials).

### 4.4. Hybridization

Hybridization of individual libraries and biotinylated baits immobilized onto streptavidin-coated magnetic particles (Sileks, Moscow, Russia) was carried out in the PCR thermal cycler at 68 °C for 48–72 h. The hybridization mixture contained blocking oligonucleotides preventing the adapter-to-adapter hybridization. Upon hybridization, the magnetic particles were washed several times in low- and high-stringency conditions, followed by washing in TE buffer (10 mM Tris-HCl, 0.1 mM EDTA, pH 8.0) at room temperature, after which individual libraries were re-amplified using primers Sol_bridge_P5 and Sol_bridge_P7 (Laboratory Protocols, Supplementary Materials) and magnetic particles as a template, with the minimum number of cycles. After purification of the PCR product with SPRI beads and measuring its concentration with BioSpec-nano (Shimadzu, Kyoto, Japan), the quality of the enrichment was verified by cloning the PCR product from twelve randomly chosen samples into the pGEM^®^-T-Easy plasmid (Promega) and sequencing of two colonies per ligation reaction. The sequences of the plasmid inserts were blasted against the Siberian fir mtDNA contigs and against GenBank database. In total, 11 plasmid sequences out of 24 corresponded to the mtDNA contigs and the Pinaceae mitochondrial sequences. One fragment was similar to *Gossipium kirkii* and the rest showed no significant match. All the 96 libraries were mixed equimolarly, the quality of the pool was checked with Agilent 2200 TapeStation System (Agilent) and sequencing was performed using the paired-end 150 bp mode with NovaSeq 6000 (Illumina) in Evrogen, Russia.

### 4.5. Data Analysis

Raw Fastq files were demultiplexed by the 8 bp indices on both sides of each fragment and preprocessed by the sequencing company. Individual sample reads were imported to the Geneious Prime^®^ 2020.2.3 software (Biomatters Ltd. Auckland, New Zealand), trimmed, paired reads were merged and duplicate reads were removed. Following error correction and normalization the reads were mapped to the reference, produced by the concatenation of the *A. sibirica* mtDNA contigs, used for the baits design (paragraph 4.1). To reduce noise from poorly sequenced regions, the minimum coverage threshold was set to 20. The homemade c++ scripts were used to clean samples from ambiguous indels and spurious nucleotide polymorphisms and to construct sequence alignment and the indel data in the 0/1 format. Alignment regions outside the reference sequence except for insertions have been removed. Due to the low quality of sequencing of the *Keteleeria fortunei* sample, mitochondrial genomes of *Picea sitchensis* [22,30], GeneBank Nos.: MK697708.1, MK697707.1, MK697706.1, MK697705.1, MK697704.1, MK697703.1, MK697702.1, MK697701.1, MK697700.1, MK697699.1, MK697698.1, MK697697.1, MK697696.1, *Larix sibirica* [23,31], GeneBank Nos.: MT797187.1, MT797188.1, MT797189.1, MT797190.1, MT797191.1, MT797192.1, MT797193.1, MT797194.1, MT797195.1 and *Keteleeria davidiana var. calcarea* (Bio-project PRJNA674712) were used as an outgroup. In the latter case, primary reads were assembled, and contigs homologous to plant mtDNA were selected using BLAST. Next, three above-mentioned mitochondrial genomes were blasted against the mitochondrial contigs of *A. sibirica*. The fragments longer than 100 bp have been mapped to the reference with Geneious and resulted sequences were added to the alignment (File S1) and edited with BioEdit [46]. Alignment length was 918895 bp.

Phylogenetic reconstruction of the genus *Abies* was conducted using maximum likelihood (ML) and maximum parsimony (MP) approach. The ML analysis was performed with RAxML v8.2 program [47] with GTR + GAMMA model and 100 fast bootstrap replicates. The MP analysis was conducted with PAUP* v. 4.0b10 [48] using heuristic search, random addition sequence with the tree bisection–reconnection (TBR) branch swapping algorithm, COLLAPSE option in effect, MaxTrees = 500 and MulTrees option in effect. Branch support was evaluated by bootstrapping using 1000 replicates.

To estimate the divergence time, a penalized likelihood approach implemented in treePL [49] was employed. The calibration was carried out similarly to [18]: the age priors were applied to four clades: (1) for Pinaceae crown group, the minimum age of 155 Mya (based on the one of the oldest Pinaceae fossils (seed cone of *Eathiestrobus mackenziei*) [50]) and the maximum age of 308.5 Mya (in accordance with unambiguous coniferous fossils in the Late Carboniferous) were used [51]; (2) for the *Abies*-*Keteleeria* divergence, the minimum age of 45.5 Mya was based on the earliest macrofossils clearly belonging to *Abies* sp. (vegetative axes, winged seeds, needles [52]) and the maximum age of 113.8 Mya corresponded to the low boundary of the Aptian age, when the oldest known fossil pollen of *Abies* was found [53,54,55]; (3) for the *Abies* crown, the minimum age of 13.5 Mya corresponded to the age of the earliest macrofossils that indubitably represent a species, related to the extant *A. bracteata* (D. Don) A. Poit (cone bracts, needles and seeds, [56]) while the maximum age was considered to be 45.5 Mya; (4) for the divergence of Pinoideae subfamily (*Larix*-*Picea* split), the minimum age of 133 Mya (the oldest record of *Pinus* [57]), whereas the maximum age of 308.5 Mya [51] were used. To estimate the divergence time, one hundred of bootstrapped ML trees with branch lengths were constructed using the maximum likelihood tree as a topology constraint in RAxML. Furthermore, using the calibration data, specified in the treePL configuration file, and having adjusted the algorithm parameters of treePL according to [58], for each bootstrapped tree the branch lengths were converted to time units. Consensus for the dated bootstrap replicates was obtained using TreeAnnotator v 2.4.7, part of the BEAST package [59].

To identify introgression between the representatives of different phylogenetic groups, we used several approaches. First, we calculated the average values of nucleotide differences and the average number of indel differences between the main phylogenetic groups, assuming that related groups, in the absence of migration from a phylogenetically distant third group, show the same level of differences with the latter, whereas in the case of migration, the differences should be decreased. Second, we used the ABBA-BABA approach [60]. This method considers four populations, i.e., two sister populations (1 and 2), a more divergent population (3) and an outgroup (4). The test checks the hypothesis of whether the gene flow from 3 to 1 had occurred. In its absence, the number of mutations common to 1 and 3 vs. 3 and 2 are expected to be equal. In case of supposed introgression of Eurasian species genes into island species (*A. sachalinensis* and *A. veitchii*) (see Results), population 1 consists of the samples of island species, population 2 combines the samples of two other American clades, while population 3 represents the Eurasian species, except for *A sachalinensis, A. veitchii* and *A. mariesii*. All groups were presented in the form of a consensus of the respective samples.

## 5. Conclusions

Based on NGS sequencing of genomic libraries of 42 *Abies* taxa enriched in mtDNA, a well-supported phylogenetic reconstruction was obtained, confirming a single main migration from the North America to Eurasia associated with seed transfer and several subsequent migrations associated with pollen transfer and hybrid capture of mtDNA of aboriginal species. The used method allowed us to identify a significant number of polymorphisms within species and between closely related species that can be used as markers of *Abies* mtDNA. The applied approach is promising for phylogenetic and population studies based on mtDNA of various plant groups.

## Data Availability

The Illumina sequence data are submitted to the NCBI SRA archive under Project Number PRJNA760249.

## Supplementary Materials

The following are available online at https://www.mdpi.com/article/10.3390/plants11060762/s1, Laboratory protocols, Table S1: List of samples used for experiments, Table S2: Position of coding regions on alignment and its representation among samples, Table S3: Average nucleotide difference (below diagonal) and average proportion of indel differences (above diagonal) among the selected groups of firs (see M&M), Figure S1. Maximum parsimony tree of mitochondrial haplotypes of *Abies*, File S1.zip: Alignment of mtDNAs of *Abies* and outgroup species, File_S2.rar: input files for RAXML.
