# Supplementary material for: Sequence Capture of Mitochondrial Genome with PCR-Generated Baits Provides New Insights into the Biogeography of the Genus Abies Mill
Source: Plants (Basel). 2022 Mar 13;11(6):762. doi: 10.3390/plants11060762 (PMC8954396; doi:10.3390/plants11060762)
Supplement: Supplementary file 1 [file plants-11-00762-s001.zip › plants-1499164-supplementary.pdf]

Supplementary materials for:

Sequence capture of mitochondrial genome with PCR - generated baits provide new insights in the biogeography of the genus Abies.

Table of Contents:

**Laboratory Protocols**	**Page 2**
**Table S1. List of samples used for experiments**	**Page 17**
**Table S2. Position of coding regions on alignment and its representation among samples**	**Page 21**
**Table S3. Average nucleotide difference (below a diagonal) and average proportion of indel differences (above a diagonal) among the selected groups of firs (see M&M)**	**Page 22**
**Figure S1. Maximum parsimony tree of mitochondrial haplotypes of *Abies***	**Page 24**

Laboratory Protocols

Preparing of baits

Target-enrichment strategies (Mamanova et al., 2010) were used to enrich NGS libraries with mitochondrial DNA. For this purpose, PCR amplified fragments of *Abies sibirica* mitochondrial DNA were used as baits. To reduce the cost and complexity of baits design, long-range PCR was employed, allowing the minimum number of primers and PCR reactions. The assembly of reads of the whole genome sequencing of *A. sibirica*, with the selection of contigs, corresponding to plant mitochondrial DNA (according to BLAST search) having the total length of about 800,000 bp was used as a template to design PCR primers.

The primers were designed using Primer3 program (Untergasser et al., 2012). Preliminary research indicated that stable amplification with a good yield of PCR product is achieved with a fragment length of up to 10,000 bp. While designing the primers, the GC content was set to at least 40%, the length of the primers was 24 bp. (see below).

Oligonucleotides used for LongAmp PCR

**Contig of *A. sibirica* mtDNA assembly**	Forward	Position on contig	sequence	Reverse	Position on contig	sequence
**1contig**	1A1F	632	CTCTCCCACACCAGAGAAGC	1A1R	11541	CCCTCAGATTGGGAAAGACA
	1A2F	11123	GCGGAGAATGTAAGCTCTGG	1A2R	22293	AATCCCCTGGGTAATTCGAC
	1A3F	21970	ATGCTTTGGTCGGTTAGGTG	1A3R	33241	TGAGAAATCCCTCCATTTCG
**2contig**	2A1F	428	GGGGAGCTGTAAAAGGAAGG	2A1R	12162	GGTCAACCAAATGTCCCATC
	2A2F	12142	GGATGGGACATTTGGTTGAC	2A2R	24022	CTCGAATAGCCGAGGACTTG
	2A3F	23766	CTGGCTCCACCAGGTGTATT	2A3R	35621	ATCTCCGCTTCAAAAGCAAA
	2A4F	35444	GAGTCTCCGAAGGCACTGTC	2A4R	46193	AATAAATCCCTTCCCCGATG
	2A5F	44572	TTTTACTGGGCCTGGAAATG	2A5R	56531	GTAGCAACGAGGGAAAGCAG
	2A6F	56478	CCTGCCTGGTAACATGGACT	2A6R	67098	TTGTCTGTCGCATTTGCTTC
	2A7F	66702	TTTATGCATGCGAACAGAGC	2A7R	79316	GAGCTTGGTGCTTGGTCTTC
**3contig**	3A1F	613	ATTATTGCTACGCCGACCAC	3A1R	11460	TGTGACAACCGGAGTACCAA
	3A2F	10824	GCAGCCAACCCCTTGTAATA	3A2R	22175	TGGCAGTGACTTCGTGAAAG
	3A3F	21983	TTTAAGCCGAGGGAAGGAAT	3A3R	33282	CATGCTCTTGCTTGTTTGGA
	3A4F	33261	CATCCAAACAAGCAAGAGCA	3A4R	45251	CGTCAAAGTGGTGGATGTTG
	3A5F	44445	GGAGCTGGCCCTTAGCTACT	3A5R	55425	GGACGTATCGATGCGAGTTT
	3A6F	55021	CCGCCAAACAAAACTACGAT	3A6R	66650	GCTTTGATGGCGCTTAGAAC
	3A7F	65773	CATTAGGCGAGTCTGGCTTC	3A7R	77416	AGAAAGGGGAATGGGAGAGA
	3A8F	76145	CTCAGAACGACGGGAAGAAG	3A8R	88000	GCATTTTGGTTTCGATCGTT
	3A9F	86644	TTTTCCCATGATCGAAGAGG	3A9R	98493	AGGAACGTAGTCGCGAAGAA
	3A10F	97380	CTCGAACCCCATACCCCTAT	3A10R	108665	CTTCGGTTGTTCCATGTGTG
	3A11F	108410	GCTGAGCCAAAACCAGCTAC	3A11R	119939	CAGAGCGAGCATTCCTTACC
	3A12F	118883	TGGATCTAACCCTTGCCTTG	3A12R	130382	TTGCTTTCAGTCCTCACACG
**4contig**	4A1F	1245	AAGTCCCGGGCTTTAACTGT	4A1R2	10595	TAGATCCTTGCTCGCACCTT
	4A1F2	10226	TGAAGCCAGCCTTTCAATCT	4A1R	20939	CCTGTAACTGCCGAAACCAT
	4A2F	20639	CGTAGGTATGCGGAACCTGT	4A2R2	28685	CCAACTCGGCTTATCTCTCG
	4A2F2	28595	GGGATCACACGACTGACCTT	4A2R	39092	AAGACAGGAGCAGCCAAAAA
	4A3F	35194	CCACCGCCTAAAGTGATTGT	4A3R2	43045	ATTTGTTAGGGCGAGGTGTG
	4A3F2	40931	TCAGACGACTCGTTCAGGTG	4A3R	51166	GGTGTCGAACGTGATTTGTG
	4A4F	51124	TCTCATTTAGCCGCTTGTCTTCAC	4A4R	64382	ACACCTGGAGCTGCTAAACTTGTG
	4A5F	62062	CCTAGTGGTTTAAGCACCCGTACC	4A5R	75583	ATCTACGTTGCACAAATTCCCTCA
	4A6F	72220	AATTGGGTCAACATCTGGTGAAGA	4A6R	86086	ATGTCGCTTGCTTATTCACTCTGC

	4A7F	83096	AGAACTGCCTCATCACCATAGCAG	4A7R	98016	ATGTTTGTAGTCAATGCCGCTTGT
	4A8F	93641	ATCTAATGGTTTTGGATGGCTCGT	4A8R	107904	AGCCGTATGAAGTGGGTTTTCATT
	4A9F	107432	GTGGTCCCCTGTTCCAAGTGTATC	4A9R	117580	ATCAATACCCGGCAGCAGAGTAAA
	4A10F	116148	AAAGTGTTCATCCGCCCTAGCATA	4A10R	127824	ATTACCTGATACTACGCGGGCTCA
	4A11F	127800	ATGAGCCCGCGTAGTATCAGGTAA	4A11R	138765	TAATATGCGACCGAAGGGAGCTAA
	4A12F	138538	TCCTTCCTGTCCTACCGATAACCA	4A12R	149991	GCAGACACAAAGTTCGCCTAGACA
	4A13F	149670	CCACTAGGCATTGTCTCACCACAC	4A13R	160909	TTGTTCAGGTTCCTCGGTTCTCTC
**5 contig**	5A1F	688	ACCAAGAACACAGCACAGAACGAC	5A1R	10608	TAAAGGCTCCCTCCTCTGAAATCC
	5A2F	10451	ATGCCCCACACTTACTCGTCGTAT	5A2R	21273	TATTTCTATGTCCCGAGGGGAGGT
	5A3F	21249	TACCTCCCCTCGGGACATAGAAAT	5A3R	33137	ACCCTCCTTATACCGGAAACCTCA
	5A4F	31277	TACCAAGCCGAACATGGTTGTATG	5A4R	42140	TTAGGATCGTTCCCATAAGCTCCA
**6 contig**	6A1F	303	GCCCCAGTTAGTTGTTCGCTATTG	6A1R	11478	ACCTTCGGAATACCCCTACAAGGA
	6A2F	9987	TCCACGGGATAAATAGAAGGAGCA	6A2R	20663	ACACCGTCCCGAGAAGAACAATAA
**7 contig**	7A1F	221	CGCAGTAAAGGTACCCACTTTTCG	7A1R	11991	TAGCTTTGGGATCCTACGATGGAA
	7A2F	11506	CGGGTTCTTAATGGATGGATGGTA	7A2R	22614	TACAACTTCCCCTCCCCTTTTGTT
	7A3F	22537	AAGGGCGTATGCCTGAAACCTTAT	7A3R	33161	GTGGAGACAGATCCAGGAAGCAAT
	7A4F	31436	GGTATCACCTTACTGCTGGCTGCT	7A4R	43392	TTATGATACCCGGGAGCACATACC
	7A5F	42390	GTGCCATAATAATGCCCACCACTT	7A5R	54296	CACTCAAGGTGCCTGTCTGTAGGA
	7A6F	53610	CCTTCTTCCATTCCCATCTTTGTG	7A7R	65515	GATTCAGGAATTTGAACCCACTGC
	7A7F	61773	CGTGGATGCGGTATACTGGGTAAT	7A7R	72223	GTCTCGAGTTCGCTTCTTCTCCAC
**8 contig**	8A1F	792	ACCGGATTCCTACTCGAACAATGA	8A1R	11338	TGCTGCGAAAGTGACCATCTCTAC
	8A2F	11132	CCTATAGCCACGCAGCTTTTTACG	8A2R	21156	GGGGCTTCCTTCGGTAACTAGAGA
**9 contig**	9A1F	533	TTCGTTCGGGTAGAGCTGTAGAGG	9A1R	11470	CCCTTGAACCCAATCATCTACTCG
	9A2F	10854	GGGAGAGGCACTTGTTCTTGTTGT	9A2R	22358	GGTATATGCGGTCCTACCAACCAA
	9A3F	20394	GCCTTATATTCTCGGCCAGTGTTG	9A3R	32200	GAGCTTTACGGTCCCTCCCTAAGA
	9A4F	31939	TAGGGTTTCTTCAGGGGTTTGGTT	9A4R	43852	ATGCCGCACCCAGAGAATATAAGA
	9A5F	42988	AGGCAGTAGTTCCTAAGGCCGTTC	9A5R	53134	AGCGAGGTTACCGCATAGTGAGAC
	9A6F	50191	CGTAAGCAAGCAGTTCATCCAATG	9A6R	57556	CTGGGCTAATCAGAGGAGGGATCT
**10 contig**	10A1F	728	TTTCCCTTGCTACCTTACGTGCAT	10A1R	10375	GGTGTGATAGGCAGGCGTTTTAAG
	10A2F	9520	GTTATCTCCTGGCTAGCGCTCGTA	10A2R	20414	ACCACTCCGGTCTCTCTCATATCG
	10A3F	19357	ATCGGATTCCCTCTGAGACTGATG	10A3R	30813	ACAGTGAAGAAGCCACCCAAGAAC
	10A4F	30525	TTACACTCCCTGCTGATGCGTTTA	10A4R	40180	GAAAGCAAGCCAGCAGGTTTAGAA
**11 contig**	11A1F	165	GTCATCTTGAGCCCTCCTGTTTGT	11A1R	10232	TTCGCCTAAGAAAGGTCTGTCCTG
	11A2F	8405	GGGTTTGTTTTCGACGGTACAGAG	11A2R	20268	ACGTGCAATACCATCTCCAACTGA
**12 contig**	12A1F	308	CGGGAGTCATTCCAGATGAGAGAT	12A1R	10728	TACACCATTCGTGCAGGTATTTGG
	12A2F	9991	CCCCAACGAAACAAAGAGTACAGG	12A2R	21902	GACTCCAATGTCTGCCTTCAGGTT
**13 contig**	13A1F	349	TAAGGGAATGATAGTTCGCCTGGA	13A1R	10094	GGTTATTTGGACACGCACGGTATT

	13A2F	9153	CGGCTATCCAGCCAAACTTACAAC	13A2R	20716	GATCCTGATCTTGCCTGAACACCT
	13A3F	19009	TAGAGGAATTCCCACCTGATCCAA	13A3R	30143	GCCGCAGAACCAATAAAAACTGAC
	13A4F	29423	ATCGATCGGACTCTGCGACTAAAC	13A4R	40796	AGAAATATGGTGCGCTAGGTTGGA
	13A5F	40286	TGCTCTCCCGTCCATTTCTCTAAG	13A5R	51361	GACCTAAAAGCTGGCCTCCAAACT
**14 contig**	14A1F	606	TGTTTGACTGCTCAATCTGCTTCC	14A1R	12374	TCTCCTTTTGGGGGATATGGAACT
	14A2F	11141	AAGGAGATCCAAAAGGGCTCGTAG	14A2R	22381	AACTGAACGCGGTGTAACCAAACT
**15 contig**	15A1F	94	ATCCCCGTCTTAGCTTATGCCATT	15A1R	11358	TCTACCAACCAGTCAGCAAGCAAG
	15A2F	10245	GCTAAGCTCCTTCAGATCCCCAAT	15A2R	21005	AGACGAAGGCACCACTCTACAACC
	15A3F	20461	TGTGTTGGGTCTCATGGATCAAGT	15A3R	28004	GATCTAGTCTGCTGGGACGGTCAT
**16 contig**	16A1F	680	TATTACCTCACGGGGAAGGGTTCT	16A1R	12223	TCCGACTTACCCTCATTACCCTCA
	16A2F	11425	AGGAAGTGTTTGACTTCGGTGGAG	16A2R	23272	GGTGTTTGAATTAGGATCGCTTCG
**17 contig**	17A1F	412	GAGTTGCGCATATTGCTGTTTCAC	17A1R	11000	AAGGGTAAGGTTGGCCAGTCAAAT
	17A2F	9557	TCAGGGTAGTCCCGGGAAAGTAAT	17A2R	21568	AAGTGTAGGTCGGACTTGGTCAGC
**18 contig**	18A1F	482	CGGCCTATATCCCTACCAATGTGA	18A1R	10334	ATAAAGCAACGGAGACATGGGCTA

Other oligonucleotides used for the preparation of baits, libraries, hybrid capture and re-amplification of libraries.

Oligonucleotide and its purpose	Sequence
Adapter oligos and primer for baits preparation	
Adapter1:	5'-Phos-GAAGCTTGAATTCGAGCAGTCAG-3'
Adapter2:	5'-CTGCTCGAATTCAAGCTTCT-3'
Bio-bait:	TEG-Bio-CTGCTCGAATTCAAGCTTCT
Adapter oligos and primers for genomic library preparation	
NEBNextad1	5Phos/GATCGGAAGAGCACACGTCTGAACTCCAGT
NEBNextad2	ACACTCTTTCCCTACACGACGCTCTTCCGATCT
Indexed primers	
**NEBNext i501 Primer**	5´-AATGATACGGCGACCACCGAGATCTACACTATAGCCTACACTCTTTCCCTACACGACGCTCTTCCGATC*T-3´
**NEBNext i502 Primer**	5´-AATGATACGGCGACCACCGAGATCTACACATAGAGGCACACTCTTTCCCTACACGACGCTCTTCCGATC*T-3´
**NEBNext i503 Primer**	5´-AATGATACGGCGACCACCGAGATCTACACCCTATCCTACACTCTTTCCCTACACGACGCTCTTCCGATC*T-3´
**NEBNext i504 Primer**	5´-AATGATACGGCGACCACCGAGATCTACACGGCTCTGAACACTCTTTCCCTACACGACGCTCTTCCGATC*T-3´
**NEBNext i505 Primer**	5´-AATGATACGGCGACCACCGAGATCTACACAGGCGAAGACACTCTTTCCCTACACGACGCTCTTCCGATC*T-3´
**NEBNext i506 Primer**	5´-AATGATACGGCGACCACCGAGATCTACACTAATCTTAACACTCTTTCCCTACACGACGCTCTTCCGATC*T-3´

**NEBNext i507 Primer**	5′-AATGATACGGCGACCACCGAGATCTACACCAGGACGTACACTCTTTCCCTACACGACGCTCTTCCGATC*T-3′
**NEBNext i508 Primer**	5′-AATGATACGGCGACCACCGAGATCTACACGTACTGACACACTCTTTCCCTACACGACGCTCTTCCGATC*T-3
**NEBNext i509 Primer**	5′-AATGATACGGCGACCACCGAGATCTACACTTGCTTGCACACTCTTTCCCTA CACGACGCTCTTCCGATC*T-3′
**NEBNext i510 Primer**	5′-AATGATACGGCGACCACCGAGATCTACACGAGAGGTTACACTCTTTCCCTA CACGACGCTCTTCCGATC*T-3′
**NEBNext i511 Primer**	5′-AATGATACGGCGACCACCGAGATCTACACACCTGGTTACACTCTTTCCCTA CACGACGCTCTTCCGATC*T-3′
**NEBNext i512 Primer**	5′-AATGATACGGCGACCACCGAGATCTACACAAGCGGAAACACTCTTTCCCTA CACGACGCTCTTCCGATC*T-3′
**NEBNext i701 Primer**	5′-CAAGCAGAAGACGGCATACGAGATCGAGTAATGTGACTGGAGTTCAGACGTGTGCTCTTCCGATC*T-3′
**NEBNext i702 Primer**	5′-CAAGCAGAAGACGGCATACGAGATTCTCCGGAGTGACTGGAGTTCAGACGTGTGCTCTTCCGATC*T-3′
**NEBNext i703 Primer**	5′-CAAGCAGAAGACGGCATACGAGATAATGAGCGGTGACTGGAGTTCAGACGTGTGCTCTTCCGATC*T-3′
**NEBNext i704 Primer**	5′-CAAGCAGAAGACGGCATACGAGATGGAATCTCGTGACTGGAGTTCAGACGTGTGCTCTTCCGATC*T-3′
**NEBNext i705 Primer**	5′-CAAGCAGAAGACGGCATACGAGATTTCTGAATGTGACTGGAGTTCAGACGTGTGCTCTTCCGATC*T-3′
**NEBNext i706 Primer**	5′-CAAGCAGAAGACGGCATACGAGATACGAATTCGTGACTGGAGTTCAGACGTGTGCTCTTCCGATC*T-3′
**NEBNext i707 Primer**	5′-CAAGCAGAAGACGGCATACGAGATAGCTTCAGGTGACTGGAGTTCAGACGTGTGCTCTTCCGATC*T-3′
**NEBNext i708 Primer**	5′-CAAGCAGAAGACGGCATACGAGATGCGCATTAGTGACTGGAGTTCAGACGTGTGCTCTTCCGATC*T-3′
**NEBNext i709 Primer**	5′-CAAGCAGAAGACGGCATACGAGATCATAGCCGGTGACTGGAGTTCAGACGTGTGCTCTTCCGATC*T-3′
**NEBNext i710 Primer**	5′-CAAGCAGAAGACGGCATACGAGATTTCGCGGAGTGACTGGAGTTCAGACGTGTGCTCTTCCGATC*T-3′
**NEBNext i711 Primer**	5′-CAAGCAGAAGACGGCATACGAGATGCGCGAGAGTGACTGGAGTTCAGACGTGTGCTCTTCCGATC*T-3′
**NEBNext i712 Primer**	5′-CAAGCAGAAGACGGCATACGAGATCTATCGCTGTGACTGGAGTTCAGACGTGTGCTCTTCCGATC*T-3′
Oligos used for hybridization and for re-amplification of libraries after hybridization	
B1	AATGATACGGCGACCACCGAGATCTACACTCTTTCCCTACACGACGCTCTTCCGATCT-Pho
B2	AGATCGGAAGAGCGTCGTGTAGGGAAAGAGTGTAGATCTCGGTGGTCGCCGTATCATT-Pho
B3	AGATCGGAAGAGCACACGTCTGAACTCCAGTCAC-Pho
B4	GTGACTGGAGTTCAGACGTGTGCTCTTCCGATCT-Pho
B5	ATCTCGTATGCCGTCTTCTGCTTG-Pho
B6	CAAGCAGAAGACGGCATACGAGAT-Pho
Sol_bridge_P5 (IS5_reamp.P5)	AATGATACGGCGACCACCGA
Sol_bridge_P7 (IS6_reamp.P7)	CAAGCAGAAGACGGCATACGA

PCR was performed using LongAmp® Taq DNA Polymerase (NEB, Ipswich, MA, USA) in 20 µL containing

H_2O	12.4 µL
5X LongAmp *Taq* Reaction Buffer	4 µL
dNTPs (10 µM)	0.6 µL
forward primer (10 µM)	0.8 µL
reverse primer (10 µM)	0.8 µL
LongAmp *Taq* DNA Polymerase	0.4 µL
Template DNA	1 µL

Genomic DNA of *A. sibirica* with a concentration of 200 ng / µL obtained earlier (Semerikova et al., 2018) was used as a template. The PCR conditions were as following: an initial denaturation at 94° C for 30 s followed by 35 cycles of 94° C for 30 s, 65° C for 45 s (for 24-nucleotide primers, or 62° C for 20-nucleotide primers) and 65° C for 12 min with the final extension at 65° C for 10 min.

The quality and relative amount of the PCR product was assessed visually by electrophoresis of 5 µL of the product conducted in 1% agarose gel in TAE buffer. Most of the fragments were amplified successfully and the product did not contain any substantial amount of non-specific amplification. The amount of the product was visually assessed by the intensity of the band (gradations 1, 2, 3, 4) and, depending on this assessment, when combined, the amount of this product was taken as 9, 6, 3, or 1.5 microliters. The total volume of the mixture was 250 µL. PCR products were cleaned using the CleanMag kit (Evrogen, Moscow, Russia) as follows:

1. 250 µL of a mixture of PCR products were combined with 0.7 volumes of magnetic particles previously resuspended and incubated for 0.5 h at room temperature (RT).

2. The tube was incubated for 10 min at RT.

3. Next, it was placed in a magnetic stand and after magnetic particles were collected (on one side of a tube) the supernatant was removed.

4. The magnetic particles were washed with 1 ml of 80% ethanol.

5. This washing step was repeated ones.

6. The remaining liquid was carefully removed and the particles were dried for 10 min under a fan.

7. Magnetic particles were resuspended in 125 µL $TE_{0.1}$ and incubated for 5 min at RT, then the tube was placed in a magnetic stand, the supernatant was transferred to a new tube and the DNA concentration was measured. It was 260 ng/µL.

Fragmentation of LongAmp PCR Products with NEBNext® dsDNA Fragmentase®

The goal was to obtain fragments less than 1000 bp, with the majority of fragments within the range of 300-600 bp.

1. Before adding the fragmentase, mix it by knocking on the tube.
2. In a 0.2 ml PCR tube, mix the following:
3. 1.5 µg LongAmp PCR product 5.7 µL
4. 10X Fragmentase buffer v2 2 µL
5. H_2O 10.3 µL
6. Fragmentase 2 µL

7. Immediately place the tube in the PCR machine at 37 °C for 15 min.

8. Quickly add 5 µL EDTA (0.5 M) to the tube and place it on ice.

9. Add 25 µL H_2O

Fractionation of the fragmentation products using CleanMag.

1. To remove long fragments, add 0.7 vol Clean Mag (35 µL), mix, incubate for 10 min at RT, place the tube in a magnetic stand and transfer the supernatant to a new tube. Discard the tube with magnetic particles.

2. To remove short fragments, add 0.25 vol CleanMag (12.5 µL), mix, incubate for 10 min at RT, place the tube in a magnetic stand and remove the supernatant. Rinse the magnetic particles with 200 µL of 80% ethanol 2 times keeping the tube in the magnetic rack, remove the residual alcohol, **do not dry!**

3. Take tube out of the magnetic stand, resuspend the particles in 20 µL H_2O. Incubate 5 min at RT, put the tube back to the magnetick rack and transfer the supernatant into a clean tube.

4. Measure DNA concentration.

End repair and A-tailing

1. Prepare adapters

In 0.2 ml PCR tube mix the following:

Adapter1 (100 µM)	20 µL
Adapter2 (100 µM)	20 µL
10mM trisHCl pH 7.0	5 µL
0.5 M NaCl	5 µL

Total volume	50 µL

Incubate the tube in PCR machine at 97° C for 2 min, 72 cycles of one min (97 ° C -1 ° C / cycle), at 25 °C for 5 min, 4 ° C ∞

2. Prepare a mixture in three tubes:

10X buffer Y (330 mM Tris-Ac (pH 7.9 at 25 °C); 100 mM MgAc; 660 mM KAc; 10 mM DTT.) (SibEnzyme, Novosibirsk, Russia)	2 µL
10 mM dNTPs	0.2 µL
100 µM ATP	0.2 µL
T4 PNK (10u/µL SibEnzyme, Novosibirsk, Russia)	1 µL
T4 DNA polymerase (5 u/µL SibEnzyme, Novosibirsk, Russia)	0.4 µL
Taq polymerase (5 u/µL SibEnzyme, Novosibirsk, Russia)	0.2 µL
Template	8 µL
Total	20 µL

Add 250 ng of DNA template to the first tube, H_2O to the second one, and 250 ng of test DNA to the third one (any purified PCR product of about 200 bp). Incubate the tube in PCR machine with the following program: 30 min at 25 °C, 30 min at 72 °C, 10 min at 4 °C.

3. Add 36 µL CleanMag (1.8 vol) to each of three tubes and clean as usual, **do not dry magnetic particles!**

4. Ligation of adapters

Prepare the mixture for each tube

10X T4 DNA ligase buffer	4 µL
50% PEG-4000	4 µL
40 µM adapter	2.5 µL
H_2O	28.5 µL
Total	39 µL

Add the mixture (39 µL) to each tube, then add 1 µL T4 DNA ligase (2,000,000 ua/ml, SibEnzyme, Novosibirsk, Russia), mix, and incubate at 22 °C for 1 h. Add 40 µL of CleanMag, clean the magnetic particles as usual, resuspend in 20 µL H_2O, incubate for 5 min at RT, and transfer the supernatant to clean tubes.

5 Amplification of baits

The products of the adapter ligation from the previous stage are used as templates. The baits are amplified in three tubes (for a higher yield), additionally, two other tubes contained as a template water or PCR product respectively.

Prepare the mixture for each of the five tubes:

H_2O	33 µL
10X Tersus Plus buffer	5 µL
dNTPS	1 µL
primer bio-Baits (10 µM)	5 µL
Tersus polymerase (Evrogen, Moscow, Russia)	1 µL
Template	5 µL
Total	50 µL

The PCR program was as follows: 94 °C, 1 min; X cycles (94 °C, 10s; 60 °C, 15 s; 72 °C 40 s); 60 °C, 15s; 72 °C, 2 min; 4 °C, ∞. It is recommended to select the number of cycles X using RealTime PCR or a series of PCR reactions with a sequential increase in the number of cycles. In our case, 24 cycles were selected. Check the PCR product on 2% agarose gel. There should be no visible product in the sample containing water as a template whereas the sample with the test PCR fragment as a template should yield the PCR product the length of which is about two adapter lengths longer than the initial one. In the target sample, PCR products should have a length distribution with a maximum of about 400 bp. Combine the contents of the first three tubes, purify with 1.5 vol. CleanMag and wash the product off the magnetic particles with 100 µL $TE_{0.1}$. Measure DNA concentration. In our case, it was 135 ng/µL. Store the baits at -20 °C.

Preparing libraries

1. Preparation of adapters

In a 0.2 ml PCR tube mix the following:

NEBNextad1 (100 µM)	20 µL
NEBNextad2 (100 µM)	20 µL
10 mM Tris-HCl, pH 7	5 µL
0.5 M NaCl	5 µL

Incubate in PCR machine with the following program: 97 °C, 2 min; 72 cycles (97 °C -1 ° C / cycle,1 min).

Store at -20 °C.

2. Fragmentation of genomic DNA

Libraries are prepared from genomic DNA (see Supporting Information, Table S1) in small batches, in our case 16 or 32 libraries, in PCR strips. Approximately 300 ng of DNA is placed in each tube. Bring the volume to 8 µL with water, add 1 µL of 10 x ds Fragmentase buffer to all tubes. On ice add dsFragmentase to the tubes using a multichannel pipette and mix the content by pipetting. Then incubate strips in a PCR machine at 37 °C for 15 min. To stop the reaction, add 2.5 µL of 0.5 M EDTA to each tube and place the strips on ice.

3. Fractionation with CleanMag to obtain fragments with an average length of about 400 bp.

Add 37.5 µL H_2O to each tube. Add 0.5 volume (25 µL) of CleanMag, mix, incubate for 10 min at RT, place tubes in a magnetic stand, and transfer the supernatant to clean tubes. Add 0.4 volume (20 µL) of CleanMag, mix, incubate for 10 min, place in a magnetic stand, remove supernatant, wash magnetic particles twice with 80% ethanol, do not dry.

4. End repair and A-tailing

Add 10 µL of the following mixture to each tube:

10X buffer Y	1 µL
10 mµM dNTPs	0.1 µL
100mM ATP	0.1 µL
T4 PNK	0.5 µL
T4 DNA polymerase	0.2 µL
Taq polymerase	0.1 µL
H_2O	8 µL

Incubate in PCR machine at 25 °C, for 30 min; at 72 °C for 30 min.

Add 10 µL H_2O, clean with 36 µL CleanMag, **do not dry!**

5. Ligation

Add to each tube 20 µL of the following mixture:

10X t4 DNA ligase buffer	2 µL
5% PEG 4000	2 µL
40 µM adapter	1.25 µL

H_2O	14.25 µL
T4 ligase	0.5 µL

Mix, incubate at 22 °C for 1 h, clean with 36 µL of CleanMag. **Do not dry!**

6. Amplification with indexing primers.

Add to each tube 47 µL of PCR cocktail prepared in advance:

H_2O	40 µL
10X Tersus Plus buffer	5 µL
10mM dNTPs	1 µL
Tersus polymerase	1 µL

Then add respective indexed primers:

NEBNext i50x Primer 10µM	1.5 µL
NEBNext i7xx Primer 10µM	1.5 µL

Run the following PCR program: 94 °C, 1 min; X cycles (94 °C, 10 s;60 °C, 15s; 72 °C, 40 s), 60 °C, 15s; 72 °C, 2 min; 4 °C, ∞.

The number of cycles X can be determined using RealTime PCR or a series of PCRs with different number of cycles within the range of 10 - 20 cycles. In our case, the number of cycles for the most of samples was 12.

Check PCR products on 2% agarose gel, purify PCR products using 1.8 vol CleanMag and dissolve in 20 µL H_2O. Measure DNA concentration.

Hybridization

1. Prepare the following mixture for each sample (in our case, multiply everything by 100):

Baits (135 ng/µL)	0.7 µL
2X BWT	2.5 µL
H_2O	1.8 µL
Total	5 µL

Split the entire volume into several PCR tubes,100 µL per tube (5 tubes in our case).

2. Incubate at 95 °C for 5 min, transfer to ice.

3. Take 4 µL multiplied by the number of samples (400 µL in our case) of streptavidin coated magnetic particles (1 µg/µL, Sileks, Moscow, Russia), mix with 1 ml 1X BWT, place in a magnetic stand, remove the supernatant.

4. Mix magnetic particles with 1 ml TET, place the tubes in a magnetic stand, remove supernatant.

5. Mix magnetic particles with baits from step 2.

6. Incubate the tubes at RT for 20 minutes.

7. Place the tubes in a magnetic stand, remove supernatant.

8. Wash magnetic particles with 1 ml 1X BWT at 65 °C, place the tubes in a magnetic stand, remove supernatant, and repeat washing step.

9. Mix magnetic particles with 125 µL (1.25 µL per tube) of TET and place the tubes on ice.

10. Prepare Library mix in PCR strips, in our case there were 12 strips. Add to each tube the following:

Blocking DNA (freshly boiled herring testes DNA 2.5 µg/µL, Sigma D6898)	0.4 µL
Blocking oligos B1-B6 (100µM) 6 X	0.25 µL
Library and H_2O	4.725 µL
Total	6.625 µL

11. Prepare the HYB buffer (multiplied by the samples number)

20X SSPE	3.125 µL
EDTA (0.5M)	0.125 µL
50 x Denhard's solution	1.25 µL
10% SDS	0.125 µL
Total	4.625 µL

Prepare with some excess. In our case, 462.5 µL

12. Run the following program on two PCR machines: 95 °C, 5 min; 72 °C, 60s; 71 °C, 60 s; 70 °C, 60s; 69 °C, 60 s; 68 °C (incubation at 68 °C can be used for plant mtDNA, but for nuclear DNA or chloroplast DNA, 65 °C should be more appropriate), ∞.

13. Place the strips with Library mix in PCR machine # 1.

14. Mix HYB buffer (462.5 µL) and magnetic particles (125 µL). Split them into 8 tubes (73.4375 µL per tube) in a PCR strip. When the temperature of the PCR block reaches 72 °C, place this strip in PCR machine # 2. Incubate for 5 min.

15. Open the strip with HYB buffer and magnetic particles mix and 4 strips with Library mix. Leave the rest of the Library mix strips closed. Resuspend the mix of HYB buffer and magnetic particles with a multichannel pipette and transfer 5.875 µL of Library mix to each tube of 4 strips, mix and layer with 10 µL of mineral oil. Close these 4 strips and start working with the next four ones etc.

16. Incubate strips with Library mix for 48 - 84 hours

17. Washing:

Add 200 µL of 1X SSC, 0.1% SDS to each tube, incubate for 10 min at RT, carefully transfer the aqueous phase with magnetic particles to clean strips, avoiding oil trapping as much as possible. Place in a magnetic stand, and remove supernatant.

Add again 200 1X SSC, 0.1% SDS to each tube, incubate for 10 min at RT, place in a magnetic stand, remove the supernatant.

Add 200 µL 0.1 X SSC, 0.1% SDS, incubate for 5 min in a PCR machine at 60 °C, remove the supernatant. Repeat this step.

Add 200 µL 0.1 X SSC, 0.1% SDS, incubate for 5 min in a PCR machine at 65 °C, remove the supernatant.

To remove traces of salts and SDS, wash magnetic particles once with 200 µL $TE_{0.1}$ at RT, remove supernatant. **Do not dry!**

18. Reamplification of libraries:

Add PCR cocktail (prepared in advance) to the tubes with magnetic particles

	Per 1 tube
H_2O	40 µL
10X Tersus Plus buffer	5 µL
dNTPs (10 mM)	1 µL
primer Sol_bridge_P5 10µM	1.5 µL
primer Sol_bridge_P7 10µM	1.5 µL
tersus polymerase	1 µL
total	50 µL

PCR program: 94 °C, 1 min; 26 cycles (94 °C, 10 s; 60 °C, 15 s; 72 °C, 40 s); 60 °C, 15s; 72 °C, 2 min; 4 °C, ∞.

Check the PCR product by conducting electrophoresis of 5 µL in 2 % agarose gel.

Purify PCR products with CleanMag (0.9 volume to reduce the proportion of short fragments). Measure DNA concentration.

19. Verification of the library enrichment by the mitochondrial DNA fragments

To check the enrichment, the PCR product of re-amplification from a part of the libraries (12 randomly taken) was ligated into the pGEM-T-Easy plasmid (Promega, Madison, WI, USA) and 2 colonies from each ligation were sequenced using the BigDye v 3.1 kit (Thermo Fisher Scientific). The sequences were blasted agains the contigs, previously used for the development of hybridization baits and against GenBank database. In our case, eleven fragments matched the contigs used for development of baits and the Pinaceae mitochondrial sequences. One fragment was similar to Gossipium kirkii and the rest showed no significant match.

20. Reamplified libraries were mixed equimolarly. The quality of the pool of the libraries was checked with Agilent 2200 TapeStation System (Agilent, Santa Clara, CA, USA) and the sequencing was done with one line of Illumina NovaSeq 6000 SP paired end 2x150 bp in Evrogen, Moscow, Russia.

RECIPIES

<u>BWT buffer (2X)</u>

2 M NaCl

10 mM Tris-Cl, pH 8.0

1 mM EDTA, pH 8.0

0.1% Tween-20

<u>BWT buffer (1X)</u>

BWT buffer (2X) diluted 2 times in water

<u>TET buffer</u>

10 mM Tris-Cl, pH 8.0

1 mM EDTA, pH 8.0

0.05% Tween-20

$TE_{0.1}$

10 mM Tris-Cl, pH 8.0

0.1 mM EDTA, pH 8.0

SSPE (20×, pH 7.4)

NaCl	3M
$NaH_2PO_4 \cdot H_2O$	0.2M
EDTA	0.02M
H_2O	to 1 L

Adjust the pH to 7.4 with NaOH. Store at room temperature.

Denhardt's reagent (50X)

Ficoll 400	1% (w/v)
Polyvinylpyrrolidone (PVP)	1% (w/v)
Bovine serum albumin (Fraction V)	1% (w/v)
H_2O	to 100 mL

20 X SSC

3M NaCl

0.3M Tris-sodium citrate

pH 7.0

Table S1 List of samples used for experiments.

sample No[1]	Species	Individual No[3]	Ratio of nucleotides read to alignment length	Indexing primer 1	Indexing primer 2	Sample information[4]	Section[5]
	Abies sibirica[2]		0.99				sect. *Balsamea*
**1**	***A. sibirica*** Ledeb.	4	0.91	i501	i701	51º48′/ 87º15′ E Is24-2007	sect. *Balsamea*
**2**	***A. sachalinensis*** (F. Schmidt) Mast.	1	0.52	i502	i701	46°40′/141°50′ E Is41-2008	sect. *Balsamea*
**3**	***A. nephrolepis*** (Trautv. ex Maxim.) Maxim.	4	0.86	i503	i701	48°15′/ 134°40′ E Is04-2008	sect. *Balsamea*
**4**	***A. alba*** Mill.	1	0.53	i504	i701	47°48′/ 13°02′ E Is6-2010	sect. *Abies*
**5**	***A. balsamea*** (L.) Mill.	3	0.44	i505	i701	MBG, voucher A0718	sect. *Balsamea*
**6**	***A. recurvata*** Mast.	2	0.67	i506	i701	S, Is15-2014	sect. *Momi*
**7**	***A. mariesii*** Mast.	4	0.41	i507	i701	35°46' / 137°48' Is5n-2015	sect. *Amabilis*
**8**	***A. firma*** Siebold & Zucc.	4	0.57	i508	i701	36°13' / 140°06' E Is7n-2015	sect. *Momi*
**9**	***A. fargesii*** Franch.	1	0.91	i501	i702	M, voucher K09-13-062 Is28-2014	sect. *Pseudopicea*
**10**	***A. amabilis*** Douglas ex J. Forbes	2	0.55	i502	i702	M, Is19-2014	sect. *Amabilis*
11	***A. bracteata*** (D. Don) A. Poit	2	-	i503	i702	NBG, Is12-2011	sect. *Bracteata*
12	***A. koreana*** E. H. Wilson	1	-	i504	i702	MBG, voucher A048 Is48-2013	sect. *Balsamea*
13	***A. pindrow*** (Royle ex D. Don) Royle	1	-	i505	i702	34°03′/ 74°24′ E Is31-2013	sect. *Momi*
**14**	***A. concolor*** (Gordon) Lindl. Ex Hildebr.	2	0.50	i506	i702	37°45′ /119°30′ W Is13-2012	sect. *Grandis*
15	***A. magnifica*** A. Murray	1	-	i507	i702	37°45′ / 119°30′ W Is16-2012	sect. *Nobilis*
16	***Keteleeria fortunei*** (A. Murray) Carrière		-	i508	i702	Is18-2017	
**17**	***A. sibirica***	1	0.92	i501	i703	49°30′/ 111°00′ E Is03-2007	sect. *Balsamea*
**18**	***A. sibirica***	5	0.91	i502	i703	52°40′/ 88°00′ Is40-2006	sect. *Balsamea*
**19**	***A. semenovii*** B. Fedtsch (synonym used in this study)	2	0.86	i503	i703	41°52′/ 71°54′ E Is33-2010	sect. *Balsamea*
**20**	***A. nephrolepis***	2	0.84	i504	i703	48°15′/ 134°40′ E Is02-2008	sect. *Balsamea* sect. *Balsamea*
**21**	***A. nephrolepis***	1	0.79	i505	i703	49°00′/ 131°05′	sect. *Balsamea*

						E<br>Is01-2008	
**22**	***A. nephrolepis***	5	0.80	i506	i703	42°51′/130°49′ E<br>Is1n-2015	sect. *Balsamea*
**23**	***A. koreana***	2	0.85	i507	i703	M,<br>voucher G01-76-1342<br>Is27-2014	sect. *Balsamea*
**24**	***A. alba***	4	0.55	i508	i703	47°48′/ 13°02′ E<br>Is8-2010	sect. *Abies*
**25**	***A. alba***	5	0.39	i501	i704	49°35′/ 19°35′ E<br>Is01-2019	sect. *Abies*
**26**	***A. cephalonica*** Loudon	1	0.54	i502	i704	B,<br>Is02-2011	sect. *Abies*
**27**	***A. cephalonica***	3	0.47	i503	i704	P,<br>Is20-2013	sect. *Abies*
**28**	***A. nordmanniana*** (Steven) Spach.	4	0.55	i504	i704	43°22′/ 41°44′ E<br>Is31-2014	sect. *Abies*
**29**	***A. nordmanniana***	1	0.51	i505	i704	B,<br>Is04-2011	sect. *Abies*
**30**	***A. nordmanniana***	3	0.41	i506	i704	S,<br>Is12-2013	sect. *Abies*
**31**	***A. nordmanniana*** ssp. ***equi-trojani*** (Asch. & Sint.ex Boiss.) Coode &Cullen	1	0.56	i507	i704	NBG,<br>Is13-2011	sect. *Abies*
32	***A. nordmanniana*** ssp. ***equi-trojani***	2	-	i508	i704	M,<br>Is23-2014	sect. *Abies*
**33**	***A. nordmanniana*** ssp. ***equi-trojani*** var. ***bornmuelleriana*** (Mattf.) Silba	3	0.60	i501	i705	M,<br>Is6-2014	sect. *Abies*
**34**	***A. pinsapo*** Boiss.	1	0.54	i502	i705	NBG,<br>Is14-2011	sect. *Piceaster*
**35**	***A. pinsapo***	3	0.53	i503	i705	P,<br>Is18-2013	sect. *Piceaster*
**36**	***A. numidica*** de Lannoy ex Carriere	2	0.57	i504	i705	NBG,<br>Is9-2013	sect. *Piceaster*
**37**	***A. firma***	5	0.65	i505	i705	36°13' / 140°06' E<br>Is8n-2015	sect. *Momi*
**38**	***A. firma***	1	0.73	i506	i705	S,<br>Is20-2011	sect. *Momi*
**39**	***A. firma***	3	0.57	i507	i705	S,<br>Is21-2011	sect. *Momi*
**40**	***A. homolepis*** Siebold & Zucc.	4	0.55	i508	i705	36°06′ / 138°11′ E<br>Is13n-2015	sect. *Momi*
**41**	***A. homolepis***	1	0.72	i501	i706	MBG,<br>voucher A0571	sect. *Momi*
**42**	***A. homolepis***	3	0.72	i502	i706	M,<br>Is25-2014	sect. *Momi*
**43**	***A. holophylla*** Maxim.	1	0.50	i503	i706	43°10′/ 132°00′ E<br>Is20-2006	sect. *Momi*
44	***A. holophylla***	3	-	i504	i706	42°05′/ 128°04′ E<br>Is22-2013	sect. *Momi*
45	***A. veitchii*** Lindl.	1	-	i505	i706	MBG,<br>voucher A0531	sect. *Balsamea*

**46**	***A. veitchii***	7	0.51	i506	i706	35°46' / 137°50' E<br>Is14n-2015	sect. *Balsamea*
**47**	***A. veitchii***	6	0.51	i507	i706	M,<br>Is5-2014	sect. *Balsamea*
**48**	***A. pindrow***	2	0.52	i508	i706	S,<br>Is14-2014	sect. *Momi*
**49**	***A. cephalonica***	2	0.62	i501	i707	NBG,<br>Is08-2013	sect. *Abies*
**50**	***A. pinsapo***	2	0.48	i502	i707	C,<br>Is30-2011	sect. *Piceaster*
**51**	***A. cilicica*** (Antoine & Kotschy) Carriere ssp. *cilicica*	1	0.46	i503	i707	Kew,<br>voucher 40673	sect. *Abies*
**52**	***A. mariesii***.	1	0.50	i504	i707	M,<br>Is01m-2014	sect. *Amabilis*
**53**	***A. mariesii***	3	0.42	i505	i707	35°46' / 137°48'<br>Is6n-2015	sect. *Amabilis*
**54**	***A. mariesii***	2	0.46	i506	i707	M,<br>voucher K09-91-132<br>Is12-2014	sect. *Amabilis*
**55**	***A. holophylla***	2	0.87	i507	i707	43°10′/ 132°00′ E<br>Is22-2006	sect. *Momi*
**56**	***A. gracilis*** Kom. (synonym used in this study)	1	0.87	i508	i707	54°07′/ 159°59′ E<br>Is51-2010	sect. *Balsamea*
**57**	***A. sachalinensis***	3	0.57	i501	i708	46°40′/143°10′ E<br>Is32-2008	sect. *Balsamea*
**58**	***A. sachalinensis***	5	0.66	i502	i708	44°01′/145°52′ E<br>Is15-2008	sect. *Balsamea*
**59**	***A. balsamea***	1	0.56	i503	i708	MBG,<br>voucher A0712	sect. *Balsamea*
**60**	***A. balsamea***	4	0.57	i504	i708	M,<br>Is9-2014	sect. *Balsamea*
**61**	***A. balsamea*** var. ***phanerolepis*** Fernald	5	0.55	i505	i708	B,<br>Is07-2011	sect. *Balsamea*
**62**	***A. fraseri*** (Pursh) Poir.	2	0.48	i506	i708	MBG,<br>voucher A0477	sect. *Balsamea*
**63**	***A. fraseri***	3	0.61	i507	i708	M,<br>voucher K09-99-143<br>Is8-2014	sect. *Balsamea*
**64**	***A. lasiocarpa*** (Hook.) Nutt.	1	0.66	i508	i708	MBG,<br>voucher A0520	sect. *Balsamea*
**65**	***A. lasiocarpa***	2	0.50	i501	i709	M,<br>K09-97-442<br>Is2-2014	sect. *Balsamea*
**66**	***A. lasiocarpa*** var. ***arizonica*** (Merriam) Lemmon	2	0.57	i502	i709	B (Otradnoye)<br>Is03-2013	sect. *Balsamea*
**67**	***A. lasiocarpa*** var. ***arizonica***	1	0.59	i503	i709	B,<br>Is06-2011	sect. *Balsamea*
**68**	***A. concolor***	4	0.41	i504	i709	37°45′ /119°30′ W<br>Is14-2012	sect. *Grandis*
**69**	***A. concolor***	1	0.42	i505	i709	B,<br>Is05-2011	sect. *Grandis*

**70**	***A. religiosa*** (Kunth) Schltdl. & Cham.	1	0.36	i506	i709	Kew, voucher 37361	sect. *Grandis*
**71**	***A. vejarii*** Martinez	1	0.45	i507	i709	Kew, voucher 37359	sect. *Grandis*
**72**	***A. grandis*** (Douglas ex D. Don) Lindl.	3	0.49	i508	i709	MBG, Is21-2013	sect. *Grandis*
**73**	***A. grandis***	4	0.50	i501	i710	M, voucher K09-03-135 Is17-2014	sect. *Grandis*
74	***A. amabilis***	1	-	i502	i710	M, Is1-2013	sect. *Amabilis*
**75**	***A. amabilis***	3	0.45	i503	i710	M, Is20-2014	sect. *Amabilis*
**76**	***A. procera*** Rehder	1	0.55	i504	i710	M, voucher K09-04-100 Is10-2014	sect. *Nobilis*
**77**	***A. procera***	2	0.54	i505	i710	M, K09-95-042 Is24-2014	sect. *Nobilis*
**78**	***A. magnifica***	2	0.51	i506	i710	37°45′ / 119°30′ W Is17-2012	sect. *Nobilis*
**79**	***A. recurvata***	1	0.28	i507	i710	Kew, voucher 36850	sect. *Momi*
**80**	***A. forrestii*** Coltm.-Rog.	1	0.58	i508	i710	Kew, voucher 37134	sect. *Pseudopicea*
**81**	***A. grandis***	1	0.59	i501	i711	Kew, voucher 40674	sect. *Grandis*
**82**	***A. nordmanniana***	2	0.59	i502	i711	MBG, voucher A480 Is25-2013	sect. *Abies*
**83**	***A. numidica***	1	0.65	i503	i711	Kew, voucher 40675	sect. *Piceaster*
**84**	***A. veitchii***	2	0.48	i504	i711	B, Is10-2011	sect. *Balsamea*
**85**	***A. spectabilis*** (D. Don) Spach.	1	0.45	i505	i711	Kew, voucher 37135	sect. *Pseudopicea*
**86**	***A. densa*** Griff.	1	0.60	i506	i711	Kew, voucher 37133	sect. *Pseudopicea*
**87**	***A. squamata*** Mast.	1	0.55	i507	i711	Kew, voucher 37136	sect. *Pseudopicea*
**88**	***A. delavayi*** Franch.	1	0.53	i508	i711	Kew, voucher 37132	sect. *Pseudopicea*
**89**	***A. gracilis***	3	0.85	i501	i712	MBG, voucher 478 Is2-2013	sect. *Balsamea*
**90**	***A. sachalinensis***	2	0.63	i502	i712	46°40′/141°50′ E Is42-2008	sect. *Balsamea*
**91**	***A. semenovii***	3	0.88	i503	i712	42°06′/ 72°49′ E Is4-2012	sect. *Balsamea*
**92**	***A. balsamea***	2	0.50	i504	i712	MBG, voucher A0715	sect. *Balsamea*
**93**	***A. concolor***	5	0.41	i505	i712	M, v. K09-00-313 Is13-2014	sect. *Grandis*
**94**	***A. durangensis*** Martinez	1	0.56	i506	i712	Kew, voucher 36849	sect. *Grandis*
**95**	***A. guatemalensis*** Rehder	1	0.50	i507	i712	Kew, voucher 7720	sect. *Grandis*

**96**	***A. bracteata***	1	0.58	i508	i712	Kew, voucher 36527	sect. *Bracteata*

[1] Sample No in sequencing experiment. Successfully sequenced indicated in bold.

[2] From Nystedt et al. (2013) (ENA accession no. ERP002568), reference used for mapping.

[3] Taxon sample number.

[4] Geographic coordinates (N/ longitude); Abbreviation of Botanical gardens and arboreta: Kew – Royal Botanical Gardens, Kew; MBG – N.V. Tsitsin Main Botanical Garden of Russian Academy of Sciences (RAS); B – Botanic Gardens of Komarov Botanical Institute RAS; NBG – Nikitsky Botanical Gardens (Yalta); P – Kornik Arboretum; S – Sochi Arboretum; C – Botanical Garden of Charles University (Prague); M – Mustila Arboretum. Sample information (voucher, isolation number) is provided.

[5] Sections are given according to the taxonomic classification of Farjon and Rushforth, 1989.

Table S2 Position of coding regions on alignment and its representation among samples.

Gene	Exon			Representation among samples, %
trnK		718	791	85
cox2	3	32538	32606	5
atp4		32950	33540	4
rps11		38847	39359	96
rps2		43784	44452	82
nad7	3	60080	60545	96
nad7	4	61612	61858	90
cob		64443	65636	92
cox2	1	74488	74869	100
rpl2	1	93478	94203	97
cox2	2	95383	95700	8
atp6		117508	118272	2
nad5	3	135465	136948	91
trnD		156953	157026	76
ccmB		190890	191528	75
nad4	4	207506	207594	92
nad4	3	210012	210434	92
nad4	2	213558	214072	99
nad4	1	230132	230593	100
ccmC		256735	257454	92
cox1		289287	290858	92
ccmFn		331184	332914	95
nad1	4	377000	377058	4
atp9		383565	383788	3
nad3		392732	393088	98
rps12		393152	393529	97
nad2	4	424016	424582	98
nad2	3	427538	427705	93
trnW		439183	439256	97
nad1	1	448467	448857	97
trnE		470136	470207	98
nad2	1	471429	471581	97
nad2	2	473085	473477	96
nad1	3	499723	499915	97
nad1	2	501643	501726	4
atp8		502342	502824	99
rps4		557462	558515	99

cox3		576007	576804	63
sdh4		576732	577168	99
trnY		593971	594053	95
trnM		597086	597161	96
nad4L		611341	611640	99
rps13		611963	612336	100
rps10	2	634212	634307	93
rps10	1	635971	636213	97
ccmFc	1	651513	652304	100
ccmFc	2	653634	654206	100
rps14		672358	672660	99
rpl5		672665	673243	98
rpl2	2	684585	685308	98
rps19		685312	685594	98
rps3	1	685598	685671	100
rps3	2	687616	687808	100
rps3	3	689496	690905	70
rpl16		690923	691336	96
trnfM		710965	711040	99
nad6		722147	722761	99
nad9		729972	730562	96
rps1		743910	744494	97
nad1	5	809944	810203	99
matR		810519	812721	96
rps7		813694	814281	91
nad7	2	822402	822470	98
nad7	1	823412	823546	95
nad5	2	837501	838733	97
nad5	1	839710	839944	98
mttB		848180	848929	97
rrn18		866570	868370	97
trnP		868635	868709	99
sdh3		868709	869153	99
nad7	5	884431	884693	97
trnM		885267	885340	85
trnQ		885929	886000	5

Table S3 Average nucleotide difference (below diagonal) and average proportion of indel differences (above diagonal) among the selected groups of firs.

	Groups								
	1	2	3	4	5	6	7	8	Outgroup
1	0.00054\ 0.02816	0.11888	0.12802	0.12417	0.13403	0.18625	0.20697	0.19123	0.53516
2	0.00200	0.00082\ 0.02482	0.09468	0.08535	0.10029	0.15677	0.18573	0.16930	0.51669
3	0.00209	0.00207	0.00040\ 0.01023	0.01853	0.09248	0.16815	0.20375	0.18981	0.54563
4	0.00190	0.00191	0.00073	0.00012\ 0.00454	0.08568	0.16490	0.19877	0.18195	0.55070
5	0.00174	0.00189	0.00176	0.00160	0.00010\ 0.00274	0.14015	0.15492	0.14645	0.52748
6	0.00182	0.00201	0.00199	0.00178	0.00166	0.00015\ 0.01233	0.10870	0.10239	0.50318
7	0.00190	0.00222	0.00219	0.00205	0.00185	0.00112	0.00026\ 0.01184	0.08601	0.51461
8	0.00194	0.00227	0.00228	0.00213	0.00188	0.00108	0.00100	0.00082\	0.49791

								0.07207	
Outgroup	0.03646	0.03695	0.04176	0.04216	0.03924	0.03712	0.03685	0.03619	0.01840\ 0.23915

Groups: 1 *A. alba, A. cephalonica, A. nordmanniana, A. pinsapo, A. numidica, A. cilicica*
2 *A. spectabilis, A. densa, A. squamata, A. delavayi, A. recurvata* (specimen *A. recurvata 1*), *A. pindrow, A. forrestii*
3 *A. sibirica, A. semenovii, A. koreana, A. fargesii*
4 *A. nephrolepis, A. gracilis, A. recurvata* (specimen *A. recurvata 2*), *A. holophylla*
5 *A. firma, A. homolepis*
6 *A. sachalinensis, A. veitchii, A. homolepis* (specimen *A. homolepis_4*)
7 *A. lasiocarpa, A. l. var. arizonica, A. balsamea, A. b. var phanerolepis, A. fraseri*
8 *A. mariesii, A. bracteata, A. amabilis, A. concolor, A. magnifica, A. grandis, A. religiosa, A. vejarii, A. procera, A. durangensis, A. guatemalensis*

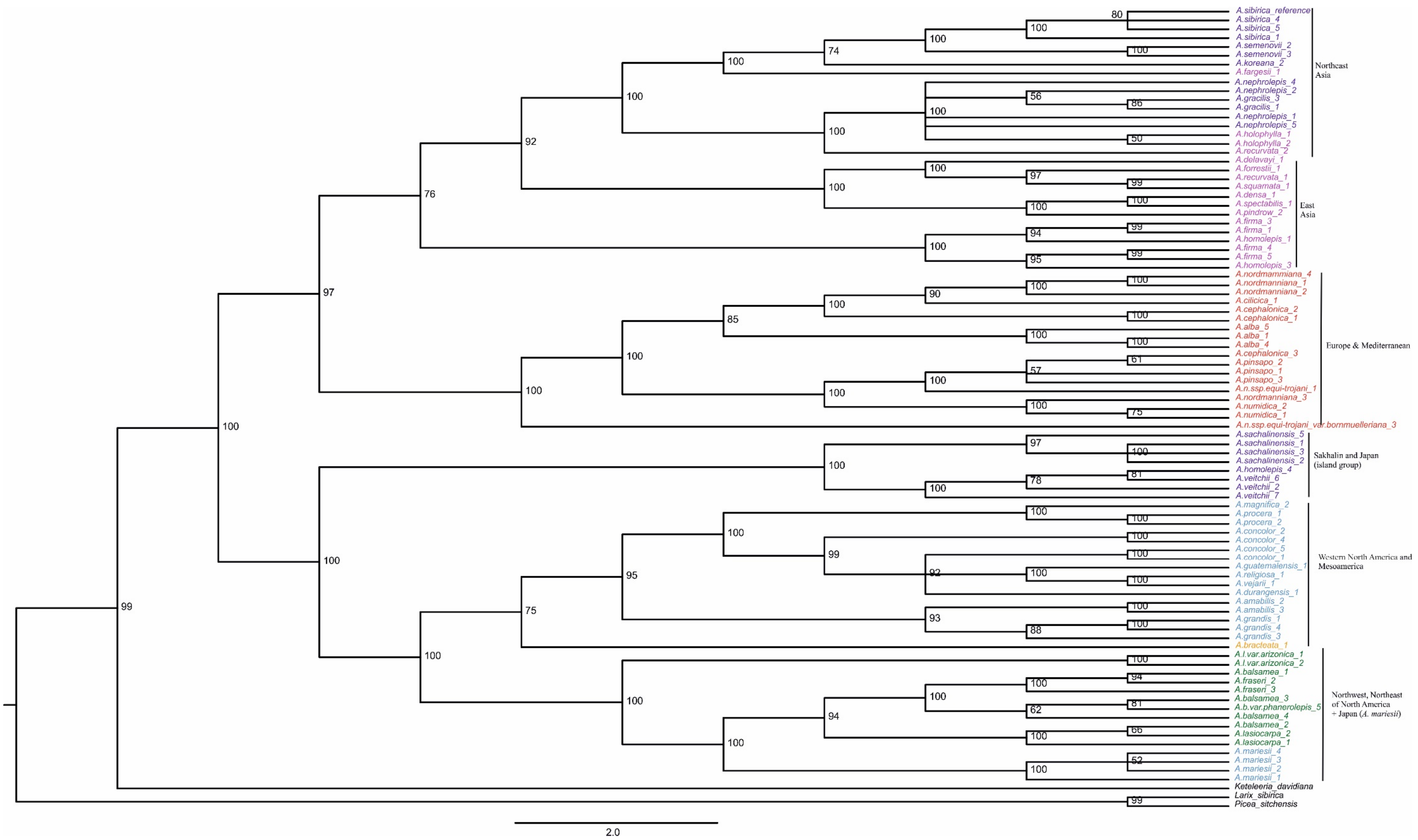

Figure S1. Maximum parsimory tree of mitochondrial haplotypes of *Abies*. Node labels represent bootstrap support. The taxon names are colored according to the nDNA-based groups (Fig. 5). Numbers following taxon names refer to taxon sample numbers (Table S1)

References

Farjon, A., Rushforth, K.D., 1989. A classification of *Abies* Miller. (Pinaceae). Notes Roy. Bot. Gard. Edinburg 46, 59–79.

Nystedt, B.; Street, N.R.; Wetterbom, A.; Zuccolo, A.; Lin, Y.C.; Scofield, D.G.; Vezzi, F.; Delhomme, N.; Giacomello, S.; Alexeyenko, A.; et al. The Norway spruce genome sequence and conifer genome evolution. Nature 2013, 497, 579-584, doi:10.1038/nature12211.

Untergasser, A.; Cutcutache, I.; Koressaar, T.; Ye, J.; Faircloth, B.C.; Remm, M.; Rozen, S.G. Primer3-new capabilities and interfaces. Nucleic Acids Res. 2012, 40, e115, doi:10.1093/nar/gks596.
